# Role of pontine sub‐laterodorsal tegmental nucleus (SLD) in rapid eye movement (REM) sleep, cataplexy, and emotion

**DOI:** 10.1111/cns.14074

**Published:** 2022-12-30

**Authors:** Fang‐Fang Fan, Ramalingam Vetrivelan, Yi Yang, Zhen‐Ni Guo, Jun Lu

**Affiliations:** ^1^ Stroke Center, Department of Neurology First Hospital of Jilin University Changchun China; ^2^ Department of Neurology Beth Israel Deaconess Medical Center and Harvard Medical School Boston USA

**Keywords:** cataplexy, fear memory, REM sleep, reward, SLD

## Abstract

Pontine sub‐laterodorsal tegmental nucleus (SLD) is crucial for REM sleep. However, the necessary role of SLD for REM sleep, cataplexy that resembles REM sleep, and emotion memory by REM sleep has remained unclear. To address these questions, we focally ablated SLD neurons using adenoviral diphtheria‐toxin (DTA) approach and found that SLD lesions completely eliminated REM sleep accompanied by wake increase, significantly reduced baseline cataplexy amounts by 40% and reward (sucrose) induced cataplexy amounts by 70% and altered cataplexy EEG Fast Fourier Transform (FFT) from REM sleep‐like to wake‐like in orexin null (OXKO) mice. We then used OXKO animals with absence of REM sleep and OXKO controls and examined elimination of REM sleep in anxiety and fear extinction. Our resulted showed that REM sleep elimination significantly increased anxiety‐like behaviors in open field test (OFT), elevated plus maze test (EPM) and defensive aggression and impaired fear extinction. The data indicate that in OXKO mice the SLD is the sole generator for REM sleep; (2) the SLD selectively mediates REM sleep cataplexy (R‐cataplexy) that merges with wake cataplexy (W‐cataplexy); (3) REM sleep enhances positive emotion (sucrose induced cataplexy) response, reduces negative emotion state (anxiety), and promotes fear extinction.

## Graphical abstract

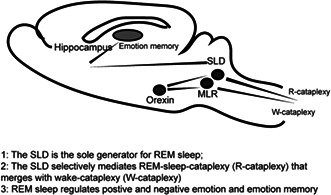

## INTRODUCTION

Animal research over last 30 years has established that the sub‐laterodorsal nucleus (SLD) in the dorsolateral pons is a key structure for REM sleep.^1^, ^2^, ^3^ For example, electrolytic or cytotoxic lesions of the SLD in rats or specific elimination of glutamatergic neurotransmission in the SLD in mice displayed reductions in REM sleep and loss of atonia (REM sleep without atonia, RSWA) and even disinhibited complex movements (REM sleep behavior disorder, RBD) such as walking, running, and jumping during REM sleep in mice and rats.^1^, ^4^ However, none of prior lesions eliminates REM sleep. Partial REM sleep loss and induction of RBD‐like movements strongly suggest incomplete SLD lesions.

Human cataplexy is mostly characterized by a sudden loss of muscle tone with intact consciousness often triggered by positive emotions such as laughter. Loss of orexin (or hypocretin) containing neurons in the lateral hypothalamus (LH) is a direct cause of human narcolepsy with cataplexy (NC).^5^ Similarly, loss of orexin neurons, peptide, or receptors caused NC symptoms in mice.^6^, ^7^ Because REM sleep‐like EEG and muscle atonia during cataplexy strikingly resemble REM sleep in mice,^8^ it is widely believed that these two states may be controlled by a common neural circuit. Consistent with this idea, activation of glutamatergic neurons in the SLD in wildtype mice induced “cataplexy”‐like state whereas their activation in 20‐25 g male orexin‐null mice (OXKO mice; mouse models of NC) increased the amounts of cataplexy. In contrast, inhibition of neither the SLD nor specific glutamatergic neurons prevented the baseline cataplexy in these mice, suggesting that cataplexy may occur in absence of the SLD.^9^ Moreover, cataplexy and REM sleep behavior disorder (RBD) may occur in the same human subjects.^10^, ^11^, ^12^ However, as chemo‐inhibition of SLD or specifically the glutamatergic neurons within the SLD hardly reduced REM sleep whereas classical neurotoxic lesions or specific elimination of glutamatergic neurotransmission did not completely abolish REM sleep.^1^, ^4^ Furthermore, SLD lesions produce RBD like movements. It suggests some SLD neurons after lesions may be functional in REM sleep generation. Thus, traditional lesion approaches cannot address whether the SLD is responsible for REM sleep and cataplexy.

REM sleep has long been proposed to be involved in the regulation of negative emotion such as fear memory and anxiety and positive emotion such as reward and addiction.^13^, ^14^ However, meta‐analysis data of a large number of REM sleep deprivation studies on anxiety in animals are inconsistent.^15^, ^16^ One factor that may cause the inconsistence is stress of various levels in sleep deprivations.

We used a recently developed viral‐based diphtheria toxin (DTA) approach with intention to completely kill SLD and eliminate REM sleep, addressing the necessary role of the SLD neurons in REM sleep, cataplexy, and emotional responses.

## THE SLD IS NECESSARY FOR REM SLEEP

To investigate whether the SLD is necessary for of REM sleep and cataplexy, we made bilateral lesions in the SLD using a diphtheria toxin‐based lesion approach and examined the changes in sleep and cataplexy in OXKO mice (male, 20‐25 g, Jackson Laboratory). For this, we injected a mixture of two AAVs—AAV containing the gene for Cre recombinase and (AAV‐Cre) and a Cre‐dependent AAV containing the gene for diphtheria toxin A (Flex‐DTA)—into the SLD of OXKO mice (*N* = 10) and implanted them with EEG/EMG electrodes. Another group of OX‐KO (*N* = 10) received sham surgery and served as the control.

**FIGURE 1:**
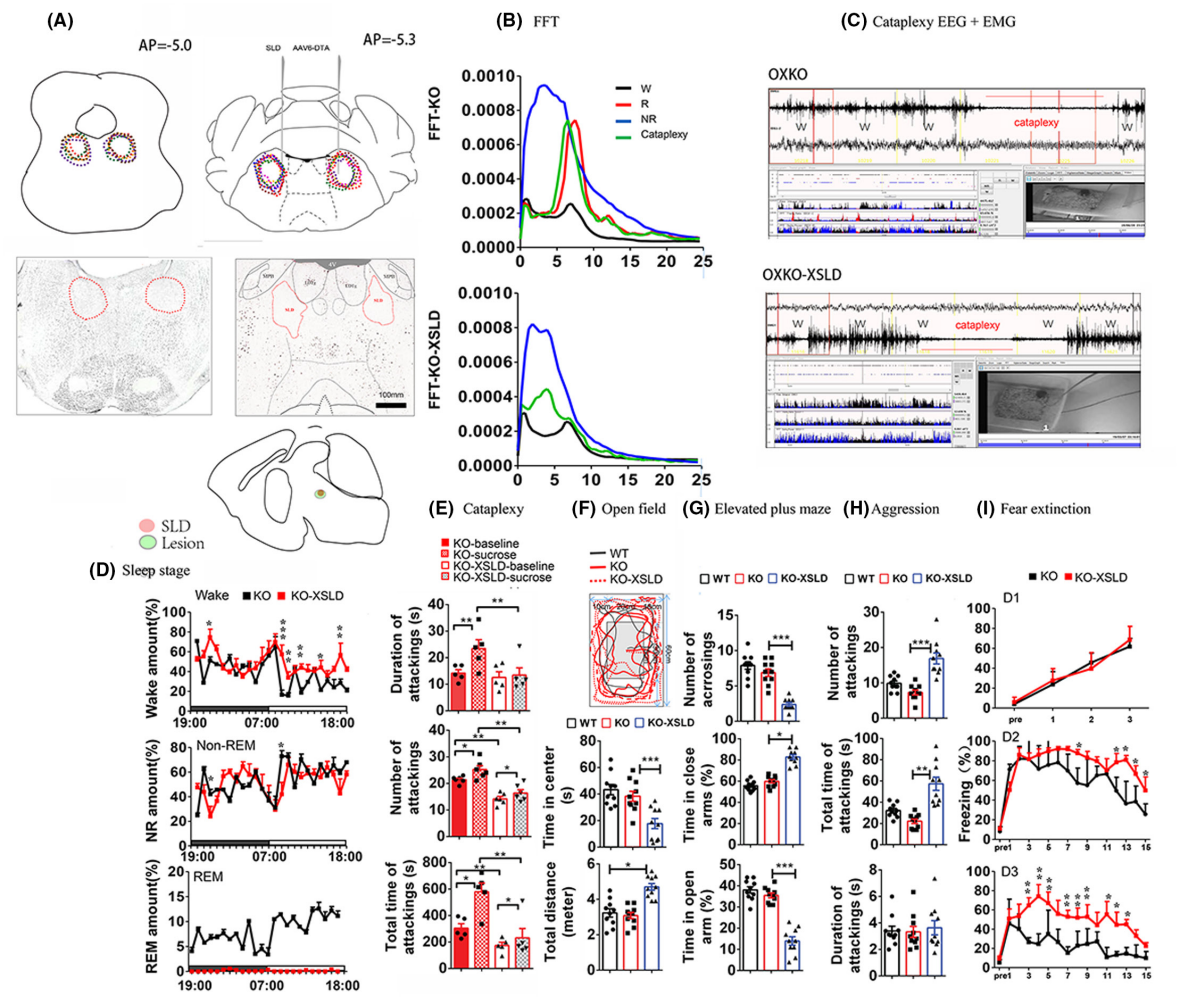
**Effects of SLD lesions on REM sleep, cataplexy, anxiety, and fear extinction.** (A) Outlines of DTA induced lesions in the SLD in all cases (upper panel) and example cases at AP = ‐5.0 and AP = ‐5.3. (middle panel) from NeuN immnostaining and SLD (red color) and lesion extension (green color) at sagittal view (bottom panel) shows bilateral lesions in the SLD (B) EEG FFT spectra of wake, REM sleep, NREM sleep, and cataplexy in OXKO mice (upper panel) and SLDx OXKO mice (lower panel) shows that baseline cataplexy FFT resembles REM sleep FFT while SLD lesions transform cataplexy REM sleep‐like FFT to wake‐like FFT. (C) A cataplexy episode in an OXKO mouse (upper panel) resembles REM sleep EEG while cataplexy in SLDx OXKO mouse (lower panel) resembles wake EEG. (D) Hourly % NEM sleep, REM sleep and wake for 24 h in OXKO controls and SLDx OXKO mice demonstrate REM sleep elimination replaced by wakefulness after SLD lesions. (E) Baseline cataplexy and sucrose induced cataplexy (bout number, total time, and bout duration) in OXKO mice and SLDx OXKO mice show that REM sleep elimination significantly reduces both basal (by about 40%) and sucrose induced cataplexy (by 75%) and sucrose induced cataplexy from 100% increase over the baseline in control to only 10% increase in SLDx. WT, OXKO and SLDx OXKO mice in OFT (E), EPM (F) and aggression test (G) show that REM sleep elimination increases anxiety‐like behaviors. (I) Fear extinction test in OXKO and SLDx OXKO mice demonstrate that REM sleep elimination significantly impairs fear extinction (D2) and extinction fear memory consolidation (D3). *p < 0.05. ** *p* < 0.01, *** *p* < 0.001.

Three weeks after the surgeries, we performed EEG/EMG with concurrent video recordings for 48 h. The lesion areas around the SLD were mapped and confirmed by NeuN staining in Figure 1A. Compared with SLD intact, the SLD lesions (SLDx) completely eliminated REM sleep and significantly increased wake amounts but did not alter NREM sleep significantly (Figure 1D). REM sleep behavior (RBD)‐like movements were not present in SLDx or control mice.

## REM SLEEP ELIMINATION REDUCES BASAL AND SUCROSE (REWARD) INDUCED CATAPLEXY WITH ALTERED FFT

EEG spectra of the baseline or sucrose induced cataplexy in OXKO controls were almost identical to REM sleep in terms of extreme high theta/delta ratio and atonia with only exception of theta peak reduced by about 1.0 Hz (REM sleep 7–8 Hz vs. cataplexy 6–7 Hz) (Figure 1B,C). After SLDx, cataplexy EEG spectra displayed wake‐like EEG with higher wide peak around 4.0 Hz than wake FFT but the overall delta power was only about a half of NREM sleep delta power (Figure 1B,C). Despite of FFT alteration after SLDx, cataplexy episodes could be clearly identified by a combination of EMG, arrest behavior (video recording) and unique FFT from active motor behaviors and high and uneven EMG in wake. SLDx OXKO mice exhibited both baseline and sucrose induced cataplexy, but their levels were significantly reduced, compared with OXKO controls (Figure 1E). SLDx reduced the baseline cataplexy amounts by about 40% time mostly from reduction in cataplexy numbers. Sucrose produced only ~10% increase in cataplexy time in SLDx OXKO mice, compared to ~100% increase in the OXKO controls (Figure 1E). Sucrose induced cataplexy that was mostly due to an increase in average cataplexy duration and less due to cataplexy number in OXKO mice was blunted by SLD lesions (Figure 1E).

## REM SLEEP LOSS INCREASES NEGATIVE EMOTION

Next, to investigate whether REM sleep elimination produced anxiety‐like behavior, we conducted open field test (OFT) and elevated plus maze (EPM) test in SLDx‐OXKO mice and OXKO mice three weeks after EEG/EMG recording. Age and sex‐matched wildtype mice (*N* = 10) were used as an additional control. In OFT, two control groups did not differ in the total travel distance or in time spent in the central area. In contrast, the SLDx‐OXKO mice traveled significantly longer total distance (restless behavior) but spent less time in the central area than both sets of controls (Figure 1F). Similarly, in the EPM SLDx OXKO mice spent significantly more time in closed arms and less time in open arms than two control groups (Figure 1G). We then assessed the aggressive behavior in SLDx‐OXKO mice, as defensive aggression is often seen in anxious animals. Consistent with the increased anxiety, SLDx mice were more aggressive than two controls. The number of attacks and the total time engaged in aggressive behavior were significantly higher in SLDx‐OXKO mice than in two controls (Figure 1H). These data indicate that REM sleep loss increases anxiety‐like behaviors.

Finally, we examined fear extinction memory in these SLDx OXKO mice and OXKO controls (*N* = 10 per group). These mice underwent a fear condition and extinction paradigm—three tones and electrical shocks in context A (light‐on and acidic acid smell) in Days 1 and 15 tones alone in context B (light‐off and pine sol smell) on Days 2 and 3. We found that SLDx‐OXKO mice showed similar fear conditioning responses in Day 1 as controls but exhibited significant higher freezing time to the tones during extinction (tone 11–15) on Day 2 and extinction memory (tone 3–15) on Day 3 than the controls (Figure 1I). These results indicate that REM sleep elimination impairs fear extinction (day 2) and memory (day 3).

## DISCUSSION

Our key findings are that SLD lesions eliminate REM sleep, reduce basal and reward induced cataplexy with EEG transformation from REM sleep‐like to wake‐like; and REM sleep elimination increases anxiety‐like behaviors and impairs fear extinction and memory.

Ever since the seminal transection studies by Jouvet and colleagues,^3^, ^17^ it was well established that the pons is necessary for REM sleep. Consequently, multiple studies employing focal lesions and stimulations identified that the sub‐coeruleus in cats or the SLD in rats is crucial for REM sleep.^1^, ^2^, ^18^ Although previous electrolytic or neurochemical lesions or genetic deletion of glutamate in the SLD resulted in REM sleep reduction by about 50% in cats, rats, and mice,^4^, ^19^, ^20^ this is the first study demonstrating complete elimination of REM sleep after SLD lesions. As REM sleep persisted albeit a lower level even after SLD lesions in previous studies, it was thought that additional REM generators might exist. Our results refute this view and prove that SLD is necessary for REM sleep. It is evident that co‐persistence of REM sleep and RBD‐like movements in those previous lesion studies are due to incomplete lesions. Given the research history of the SLD, despite data were obtained from the OXKO mice, the lesioned results are likely seen in WT mice and rats.

Given highly similarity of EEG FFT and atonia in cataplexy and REM sleep in OXKO mice, cataplexy is proposed to be abnormal REM sleep intrusion into wakefulness. Our detailed FFT analysis indicated that cataplexy FFT theta peak was about 1.0 Hz slower than that of REM sleep FFT. After REM sleep elimination, there was 40% reduction in cataplexy amounts with wake‐like EEG peaking around 4.0 Hz that significantly differs from cataplexy REM‐like FFT in OXKO, which suggests that cataplexy in OXKO mice may be a mixed state of wake‐onset REM sleep (R‐cataplexy, 7–8 Hz theta EEG) and wake‐onset atonia (W‐cataplexy, 4 Hz EEG). As R‐cataplexy has much higher power in theta EEG than W‐cataplexy 4 Hz, two state merging may produce 6–7 Hz theta EEG. If cataplexy is indeed a mixed state, one may predict that direct single cell recording in the hippocampus in OXKO mice would show identical firing patterns in REM sleep and cataplexy. W‐cataplexy overall delta power was higher than wake delta power but only about 50% delta power of NREM sleep, thus it is a distinct atonia state. Similar EEG pattern with high 2–4 Hz originated from the frontal cortex has been reported in freezing behavior in mice and cataplexy‐like behavior by the mesencephalic locomotor region (MLR) lesions in rats.^21^, ^22^, ^23^ Rats of orexin‐ataxin that produces incomplete loss of orexin neurons exhibit wake‐like FFT cataplexy.^24^ W‐cataplexy as a conscious state may well be due to incomplete loss of orexin neurons. Small amounts of orexin may be sufficient to prevent R‐cataplexy. We predict that human with the extreme low‐level orexin displays R‐cataplexy intrusions with rapid eye movements and dream‐like hallucination into W‐cataplexy. Indeed, there is a case report that REM‐sleep like eye movements and dreamlike hallucination intrusion into cataplexy.^25^ Regarding the neural circuit of W‐cataplexy, our previous studies suggest that the MLR reticulospinal neurons that contain orexin 2 receptors may mediate W‐cataplexy as MLR lesions produce cataplexy‐like behavior with a high delta wake EEG in rats.^21^, ^26^ It is not clear how two distinct states, R‐cataplexy and W‐cataplexy, mix up in cortical activity. One possibility is that they merge at a small time scale, so that EEG FFT appears to be evenly and continuous theta dominant, producing hallucination (REM sleep) and awareness (wake) simultaneously. We hypothesize that orexin system through the SLD and MLR simultaneously prevents R‐cataplexy and W‐cataplexy.

Selective REM sleep elimination by SLD lesions enabled us to investigate the role of REM sleep in regulation of positive and negative emotion in stress‐freely model. The role of REM sleep in enhancing reward (sucrose) induced cataplexy is in consistent with the study that REM sleep amounts correlate to reward rating in human.^14^ By contrast, REM sleep loss increased anxiety‐like behaviors in OFT and EPM. In addition, restless locomotion in OFT and aggressive behavior after REM sleep elimination is line with restlessness and increased aggression in attention‐deficit/hyperactivity disorder (ADHD).^27^ Finally, we found that REM sleep elimination impaired fear extinction and memory, which again indicates that REM sleep reduces fear emotion and memory by enhancing fear extinction and consolidating fear extinction memory. Thus, REM sleep may regulate emotions by reducing negative emotions and elevating positive emotions.

Meta‐analyses on associations between REM sleep and anxiety using sleep deprivation in a large number of animal findings are inconsistent.^15^, ^16^, ^28^ We speculate that inconsistent and various level stress by sleep deprivation methodology produces different outcomes on anxiety and fear emotion, which may cause the inconsistent outcomes. It appears that human sleep deprivation and insomnia consistently display high anxiety,^16^ while sleep deprivations have inconsistent effects on in fear memories in both animals and human.^13^ Although our animal experiment could not resolve whether REM sleep regulates conscious vs subconscious emotion and memory, a recent study of human sleep apnea occurred during REM sleep or during NREM sleep suggests that REM sleep may particularly regulate subconscious negative emotion and memory.^29^

Our results suggest that REM sleep regulates cataplexy in two aspects: being a part of cataplexy and emotion perception. REM sleep loss would remove R‐cataplexy and blunt emotion.

In conclusions: the SLD is the sole generator for REM sleep and responsible for R‐cataplexy; REM sleep increases positive emotion and reduces negative emotion and memory.

## CONFLICT OF INTEREST

The authors declare no conflict of interest.

## Supporting information


Appendix S1.

## Data Availability

The data that support the findings of this study are available from the corresponding author upon reasonable request.
