# Supplementary material for: Role of pontine sub‐laterodorsal tegmental nucleus (SLD) in rapid eye movement (REM) sleep, cataplexy, and emotion
Source: CNS Neurosci Ther. 2022 Dec 30;29(4):1192–6. doi: 10.1111/cns.14074 (PMC10018081; doi:10.1111/cns.14074)
Supplement: Supplementary file 1 — Appendix S1. [file CNS-29-1192-s001.docx]

**Methods and Results**

**SLD lesions**: 20-25g male OXKO mice (N=10, Jackson Laboratory) were anaesthetized with ketamine/xylazine (80mg/kg/6mg/kg) and placed into a stereotaxic frame. We placed bilateral injections of a mixture of an AAV vector expressing diphtheria toxin A subunit (rAAV8-EF1α-mCherry-Flex-dtA; 3.7×10^12^ viral particles/ml, University of North Carolina Vector Core ) and AAV8-cre mixture (100 nlL, University of North Carolina Vector Core) 100 nl per side at AP = - 5.3 mm, ML = ± 0.1mm, DV = - 3.2mm into the SLD of OXKO mice. This vector expresses mCherry in all transfected neurons, but kills neurons expressing cre-recombinase. The injections were performed using glass pipettes with a 10 to 20 μm diameter tip and a pressure-injection system. After injections, mice were implanted with four EEG (electroencephalography) screw electrodes (two frontal and two parietal electrodes; Pinnacle Technology Inc.) and two ﬂexible EMG (electromyography) wire electrodes (Plastics One) previously soldered to a 6-pin connector (Heilind Electronics, Inc.) and the assembly was secured with dental cement (Lang Dental Mfg. CO., Inc, Wheeling, IL) and wound was closed. 10 OXKO mice received sham surgery were used for control.

Three weeks after surgery, SLDx and control OXKO mice were housed individually in transparent barrels in an insulated sound-proofed recording chamber maintained at an ambient temperature of 22.0±1.0°C and on a 12-h light/dark cycle (lights-on at 07:00) with food and water available ad libitum. Mice were habituated to the recording cable for 3 days before starting EEG/EMG/video recording for 48 hours. Three behavioral states (Wake, NREM and REM sleep) were visually identified and analyzed in 10-s epochs using SleepSign for Animal (Kissei, Japan). Fast Fourier Transform (FFT) was calculated for wake, NREM sleep, REM sleep, cataplexy separately. Cataplexy episode must satisfy the followings: sudden arrest with low and smooth EMG during active wakefulness with REM sleep like theta EEG or high 4 Hz wake-like EEG at least 10 seconds. As mice had low level of active-wakefulness during day time, we only determined cataplexy attacks during the dark period (19:00-7:00). For reward inducing cataplexy, we presented two bottles of 10% sucrose solution and water during the night.

**Histology**: Mice were perfused by formalin. The frozen brains were sectioned at 40 micrometer in two series. The sections were immunolabeled with NeuN (1:10K, Abcam). The areas that were absence of NeuN staining were lesioned.

**Open field test**: A mouse was placed in an arena (50 cm x 50 cm) with walls (38 cm) for 5 mins. By tracking travel distance and time spent in central (40cm by 40cm) and peripheral areas from recorded video, we calculated total travel distance, time in the center and the peripheral area.

**Elevated plus maze test**: The apparatus comprises two open arms (25 x 5 x 0.5 cm) across from each other and perpendicular to two closed arms (25 x 5 x 16 cm) with a center platform (5 x 5 x 0.5 cm). The time spent for each mouse in each arm, and entries into the open arms for total 5 minutes were calculated.

**Aggression test**: A resident male SLDx or control mouse in the homecage was introduced to a novel intruder male mouse for 5 minutes. Aggression attack numbers and durations were measured based on the recorded video.

**Fear memory test:** We used a Med Associates system for testing and analysis. On day 1, four mice were placed in four separated boxes with interior white light and 1.0% acetic acid (context A) for 180 seconds, and exposed to the first tone (18 seconds, 2000Hz, 85 dB) and electrical shock (2 seconds, 0.54 V), then a 30 seconds interval (I1), followed by second and third tone and shock. On day 2 and 3, mice were placed in the same boxes with light-off context and pine oil smell (context B) for 120 seconds pre-tone period and then 15 tones with 20 seconds duration separated by 30 seconds intervals. Freezing time was measured as the percentage of observations below the threshold by video recording.

**Statistical tests**: Shapiro-Wilk confirmed the Gaussian distribution of all data (W<1.96, p>0.05). ANOVA and Bonferroni’s post-hoc test by SPSS 19.0 (IBM Corp, Armonk, NY, USA) were used to detect the differences in sleep analysis and behavior tests between SLDx OXKO mice and OXKO control mice. In anxiety tests, WT as additional control group was compared to OXKO and SLDx OXKO mice. The data were presented as mean ± standard error.
